# A Case of Anchylosis of Both Elbow Joints, Treated by Forced Rupture of the Adhesions

**Published:** 1858-10

**Authors:** J. W. Freer

**Affiliations:** Prof. of Anatomy in Rush Medical College


### ARTICLE IV.

### A CASE OF ANCHYLOSIS OF BOTH ELBOW JOINTS, TREATED BY FORCED RUPTURE OF THE ADHESIONS.

BY J. W. FREER, M. D., PROF. OF ANATOMY IN RUSH MEDICAL COLLEGE.

CASE.—Patrick Flynn, aged forty-five years, residing in the Alms House; appearance, healthy and robust.

*History.*—On the 15th of December, 1857, fell from a second story window to the pavement, dislocating both elbow joints, and fracturing both of the internal condyles. The fact of the dislocations was established merely from the statement of the patient; that of the fractures was apparent at the time he came under my care, February 23d, 1858.

*Present Appearances.*—Both limbs in the straight position, and very nearly immovable; the left ulnar nerve paralyzed. The anchylosis being of that variety termed false, the patient was unable to touch any portion of the head or face with his hands. His utensils for feeding or drinking were provided with handles corresponding to the length of his arms.

We can readily imagine the peculiar unpleasantness of such a condition, and the strong claims it presented to surgical science for relief. I therefore resolved to treat the case by the modern invention of rupturing the adhesions by direct force.

*Operation.*—The patient was put fully under the influence of chloroform, preparatory to the operation. Then selecting the right limb, and placing my knee over the bend of the elbow, and making use of the forearm as lever, the rupture was accomplished speedily and without difficulty, so that flexion and extension could be produced very nearly to the full extent.

*Subsequent Treatment.*—The limb was placed at right angles and supported in a sling, and evaporating lotions ordered.

Slight inflammatory action followed, which subsided in three or four days, when passive motion was made use of until the usefulness of the member was restored.

*Feb. 29th.*—The opposite arm was operated on, and treated in the same manner as the first, and with the same results.

On May 29th, when I last saw the patient, the usefulness of the limbs was restored to the extent of feeding himself with ordinary utensils, and of being able to perform various duties about the Alms House.

For the history of this method of treating anchylosis, I would refer the reader to an article of mine in the *N. W. Medical and Surgical Journal* in 1856.

